# Dissection of the Genetic Basis of Yield Traits in Line per se and Testcross Populations and Identification of Candidate Genes for Hybrid Performance in Maize

**DOI:** 10.3390/ijms23095074

**Published:** 2022-05-03

**Authors:** Yuting Ma, Dongdong Li, Zhenxiang Xu, Riliang Gu, Pingxi Wang, Junjie Fu, Jianhua Wang, Wanli Du, Hongwei Zhang

**Affiliations:** 1 Agronomy College, Shenyang Agricultural University, Shenyang 110866, China; 15140085901@163.com; 2 National Key Facility for Crop Gene Resources and Genetic Improvement, Institute of Crop Sciences, Chinese Academy of Agricultural Sciences, Beijing 100081, China; 15993216250@163.com (D.L.); wangpingxi2009@163.com (P.W.); fujunjie@caas.cn (J.F.); 3 Center for Seed Science and Technology, College of Agronomy and Biotechnology, China Agricultural University, Beijing 100193, China; dlxuzhenxiang@163.com (Z.X.); rilianggu@cau.edu.cn (R.G.); wangjh63@cau.edu.cn (J.W.)

**Keywords:** maize, testcross, yield per plant, hundred kernel weight, candidate gene

## Abstract

Dissecting the genetic basis of yield traits in hybrid populations and identifying the candidate genes are important for molecular crop breeding. In this study, a BC1F3:4 population, the line per se (LPS) population, was constructed by using elite inbred lines Zheng58 and PH4CV as the parental lines. The population was genotyped with 55,000 SNPs and testcrossed to Chang7-2 and PH6WC (two testers) to construct two testcross (TC) populations. The three populations were evaluated for hundred kernel weight (HKW) and yield per plant (YPP) in multiple environments. Marker–trait association analysis (MTA) identified 24 to 151 significant SNPs in the three populations. Comparison of the significant SNPs identified common and specific quantitative trait locus/loci (QTL) in the LPS and TC populations. Genetic feature analysis of these significant SNPs proved that these SNPs were associated with the tested traits and could be used to predict trait performance of both LPS and TC populations. RNA-seq analysis was performed using maize hybrid varieties and their parental lines, and differentially expressed genes (DEGs) between hybrid varieties and parental lines were identified. Comparison of the chromosome positions of DEGs with those of significant SNPs detected in the TC population identified potential candidate genes that might be related to hybrid performance. Combining RNA-seq analysis and MTA results identified candidate genes for hybrid performance, providing information that could be useful for maize hybrid breeding.

## 1. Introduction

With the increase in human population, it is expected that the production of staple food crops should be doubled to feed the growing population by 2050 [1]. Hybrid crop breeding can increase crop yield and meet human demand for food. As a successful example of hybrid breeding, hybrid maize has played an important role in maize yield increase in the last century [2]. To further increase the yield of hybrid maize by using molecular design breeding, it is necessary to dissect the genetic and molecular mechanism of hybrid performance in hybrid populations.

In maize, hybrid breeding generally requires selection of breeding materials according to line per se (LPS) and testcross (TC) performance [3]. However, LPS performance can only indirectly assess TC performance [4], and the accuracy of selection based on LPS performance depends on the relationship between the LPS and TC population. The genomic differences between an LPS population and its TC counterpart are the replacement of one half of its genome by the genome of the tester. Thus, the masking effect of the dominant alleles from the tester and epistasis caused by the tester genome might influence the correlation between the performance of the LPS population and that of the TC population [5,6]. Comparison of the genetic basis of the LPS and TC populations would unravel the differences between the two populations and help us understand their correlation.

Marker–trait association (MTA) analysis identifies the association between the tested traits and molecular markers within a population. To date, many MTA analysis methods have been developed, such as the general linear model, mixed linear model, and FarmCPU (fixed and random model circulating probability unification) [7,8,9,10], but most methods can only be used for MTA analysis in populations containing inbred lines. Because there are both homozygous and heterozygous genotypes in the genotypic data of hybrid populations, statistical methods and models that include both dominant and additive effects should be developed. Huang et al. (2015) used different encoding schemes to detect the genetic basis of additive, dominant and recessive effects [11]. Another study used a linear mixed model fitting both additive and dominance effects to detect additive and dominant quantitative trait locus/loci (QTL) [12]. Zhang et al. [13] provided a user-friendly pipeline for detecting QTL in hybrid populations. The pipeline considered additive, dominance and epistatic effects and used likelihood ratio test (LRT) statistics to declare the statistical significance. These models have been proven reliable for performing MTA analysis in hybrid populations.

Various methods provide evidence for the selection of candidate genes in QTL regions. RNA sequencing (RNA-seq) was able to identify differentially expressed genes (DEGs) among multiple samples or treatments and has been frequently used to find candidate genes controlling various traits [14,15,16]. Gene annotation information was also used to identify and confirm the candidate genes within a chromosomal interval. By analyzing the DEGs in the mapping interval, the candidate gene controlling maize glossy phenotype was identified and functionally characterized [14]. The candidate genes for a plant height QTL were selected based on RNA-seq analysis and gene annotation information [15]. Taken together, RNA-seq and gene annotation analysis could be used to select candidate genes in QTL regions.

In this study, we constructed a BC1F3:4 population by using elite inbred lines Zheng58 (donor parent) and PH4CV (recurrent parent). The population was testcrossed to Chang7-2 and PH6WC. The three populations were evaluated for hundred kernel weight (HKW) and yield per plant (YPP). Additionally, we performed RNA-seq analysis using two widely used hybrid varieties and their parents to find the DEGs between hybrids and their parental lines. The objectives of this study were to dissect the genetic basis of HKW and YPP in the three populations and to assess the genetic features of the significant SNPs. We also found the DEGs between hybrid varieties and their parental lines and identified the candidate genes by comparing the locations of the significant SNPs and DEGs.

## 2. Results

### 2.1. Phenotypic Data of the Three Populations

**Figure 1:**
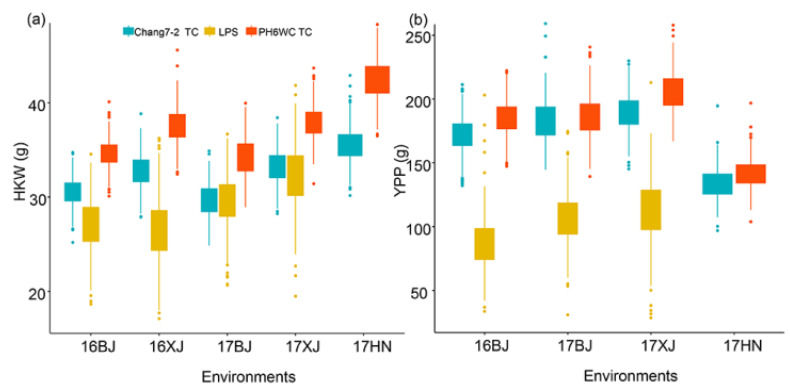
Boxplot showing the distribution of HKW and YPP of the three populations. (**a**) and (**b**) Distribution of HKW and YPP, respectively.

The PH6WC TC population had the highest HKW and YPP across all the environments (Figure 1, Table 1), followed by the Chang7-2 TC population, suggesting that PH6WC generally had better combining ability with the LPS population than Chang7-2. Both traits of the LPS population had a higher coefficient of variance and broad-sense heritability, indicating that the genetic variation was greater and more stable than that of the two TC populations. Furthermore, HKW had higher heritability than YPP, indicating that HKW was more stable than YPP across environments (Table 1). The correlation of either HKW or YPP across environments was generally significant for each population (Figure S1), suggesting that the genetic basis plays a major role in determining the two traits across environments.

### 2.2. Genotypic Data Analysis and Genetic Dissection of Yield Traits of the Three Populations

**Figure 2:**
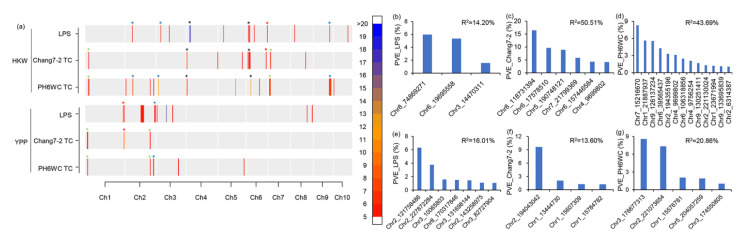
Distributions and effects of significant SNPs. (**a**) Distribution of significant SNPs controlling each trait of each population; the colors show the density of significant SNPs within a 1 Mb interval; red asterisk indicates common QTL between LPS and Chang7-2 TC populations; blue asterisk indicates common QTL between LPS and PH6WC TC populations; green asterisk indicates common QTL between Chang7-2 TC and PH6WC TC populations; dark asterisk indicates common QTL among LPS, Chang7-2 TC and PH6WC TC populations. (**b**–**d**) PVE of the large-effect SNPs controlling HKW of the LPS, Chang7-2 and PH6WC TC populations, respectively. (**e**–**g**) PVE of the large-effect SNPs controlling YPP of the LPS, Chang7-2 and PH6WC TC populations, respectively. In (**b**–**g**), only SNPs with PVE larger than 1% are shown; PVE_LPS, PVE_Chang7-2 and PVE_PH6WC indicate phenotypic variance explained by the significant SNPs in the LPS, Chang7-2 and PH6WC TC populations, respectively.

In total, 15,386 SNPs were obtained after genotype processing, and these SNPs were distributed evenly across the maize physical map. The number of SNPs ranged from 1056 on chromosome 10 to 2477 on chromosome 1, and the SNP density ranged from 6.62 SNP/Mb on chromosome 2 to 8.80 on chromosome 9, with a mean density of 7.5 SNP/Mb (Table S1). The SNP density was sufficiently high for MTA analysis [17]. MTA found that the numbers of significant SNPs for HKW were 24, 30 and 121 in the LPS population, Chang7-2 and PH6WC TC populations, respectively (Table S2). These SNPs were distributed on all chromosomes (Figure 2a). The top large-effect SNPs controlling HKW of the LPS population were on chromosomes 2, 3, 4, 5, 6, 8, 9 and 10 (Figure 2b), whereas those of the Chang7-2 TC population distributed on chromosomes 1, 4, 6, 5, 7 and 8 (Figure 2c), and those of the PH6WC TC population distributed on chromosomes 1, 2, 3, 4, 5, 6, 7, 9 and 10 (Figure 2d). The significant SNPs for HKW totally explained 14.20%, 50.51% and 43.69% of phenotypic variance in the LPS population, Chang7-2 and PH6WC TC population, respectively (Figure 2b–d).

The number of significant SNPs for YPP was 151, 27 and 29 in the LPS population, Chang7-2 and PH6WC TC populations, respectively (Table S2). These SNPs mainly distributed on chromosomes 1, 2, 3, 5 and 8 (Figure 2a). The top large-effect SNPs controlling YPP of the LPS population were on chromosomes 2, 3 and 8 (Figure 2e), whereas those of the Chang7-2 TC population were on chromosomes 1 and 2 (Figure 2f), and those of the PH6WC TC population were on chromosomes 1, 2, 3 and 5 (Figure 2g). The significant SNPs for YPP totally explained 16.01%, 13.60% and 20.86% of phenotypic variance in the LPS, Chang7-2 and PH6WC TC population, respectively (Figure 2e–g).

### 2.3. Genetic Features of the Significant SNPs

**Figure 3:**
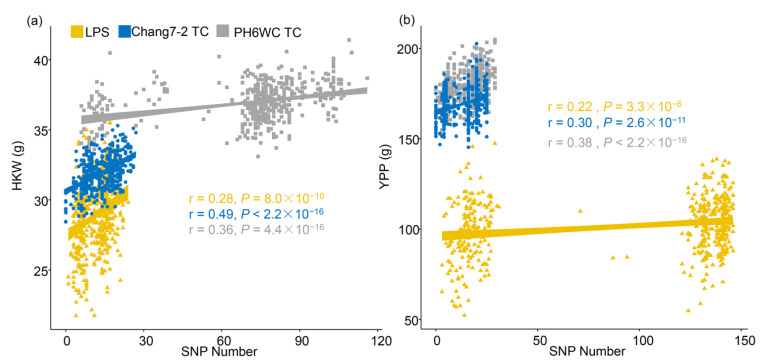
HKW and YPP increased with the increase of the number of favorable genotypes of significant SNPs. (**a**) showed that HKW increased with the increase of the number of favorable genotypes of significant SNPs in the LPS (yellow), Chang7-2 TC (blue), and PH6WC TC (gray) populations; (**b**) showed that YPP increased with the increase of the number of favorable genotypes of significant SNPs in the LPS (yellow), Chang7-2 TC (blue), and PH6WC TC (gray) populations.

We calculated the cumulative effects of favorable genotypes of the significant SNPs. The correlation coefficients between the number of favorable genotypes and HKW were 0.28, 0.49 and 0.36 for the LPS, Chang7-2 and PH6WC TC populations, respectively (Figure 3a). The correlation coefficients between the number of favorable genotypes and YPP were 0.30, 0.22 and 0.38 for the LPS, Chang7-2 and PH6WC TC populations, respectively (Figure 3b). The strong positive correlation indicates that the LPS performance and TC performance increased with the accumulation of favorable genotypes, which further proved the reliability of the MTA results.

**Figure 4:**
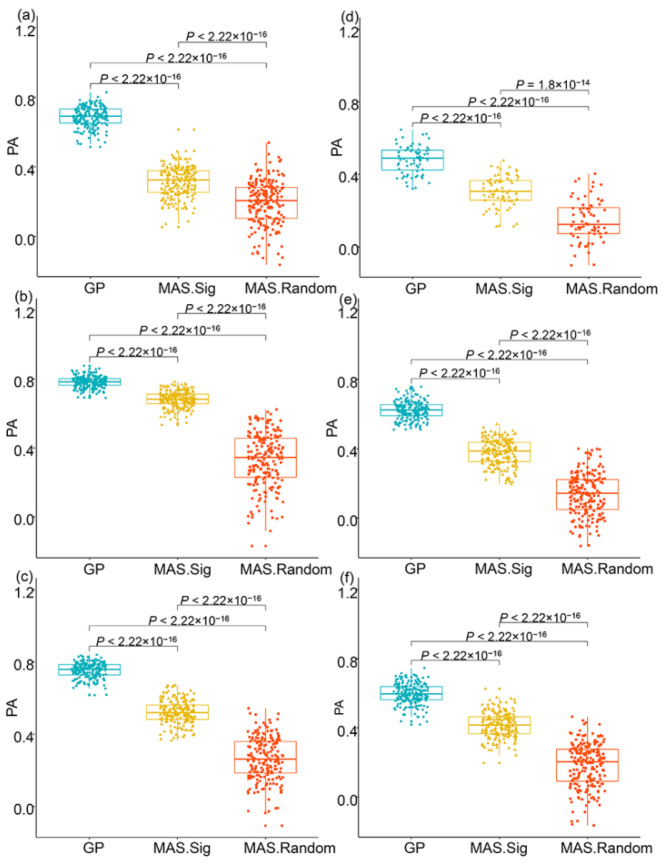
Using the significant SNPs to predict phenotypes in each population. (**a**–**c**) PAs of the GP, MAS.Sig and MAS.Random model in predicting HKW in the LPS (**a**), Chang7-2 TC (**b**) and PH6WC TC (**c**) populations, (**d**–**f**) PAs of the GP, MAS.Sig and MAS.Random models in predicting YPP in the LPS (**d**), Chang7-2 TC (**e**) and PH6WC TC (**f**) populations. GP indicates GP models using all SNPs; MAS.Sig indicates MAS models using the significant SNPs; MAS.Random indicates MAS models using the same number of randomly selected SNPs. *p* values indicate significant levels of Wilcox.test.

To test the effect of these significant SNPs in predicting LPS and TC performance, we performed genomic prediction (GP) and marker-assisted selection (MAS) analysis. The analysis revealed that the prediction accuracies (PAs) of GP models were larger than those of the MAS.Sig model (Figure 4a–c), suggesting that some genetic factors were not identified due to the problem of false negatives for both traits in each population. We also found that the PAs of the MAS.Sig model were larger than those of MAS.Random model (Figure 4d–f) for each trait in each population, further proving that the significant SNPs were in linkage disequilibrium with the genes controlling the tested traits.

### 2.4. Identification of Common QTLs between LPS and TC Populations

**Figure 5:**
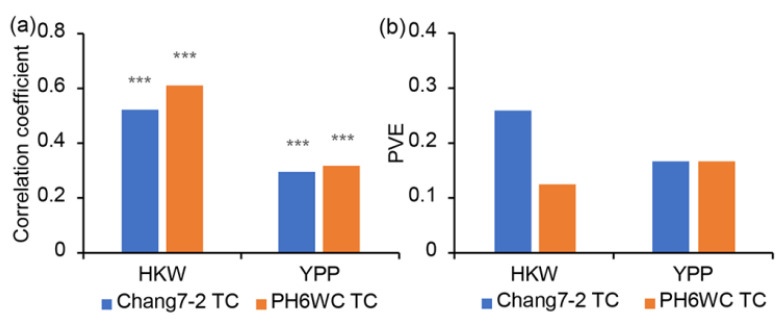
The relationship between LPS and TC populations. (**a**) Correlation coefficients between LPS and each of the two TC populations; *** indicates significance at 0.001 level. (**b**) Phenotypic variance of TC populations explained by the significant SNPs detected in the LPS population.

Given the significant correlation between the tested traits of the LPS population and the TC population (Figure 5a), we considered that there should be common QTLs controlling LPS and TC performance. To prove this hypothesis, we examined whether the significant SNPs of the LPS population take effect in the TC populations. The results showed that the significant SNPs for HKW of the LPS population explained 25.82% and 12.24% of phenotypic variance in the Chang7-2 and PH6WC TC populations, respectively (Figure 5b). Furthermore, the significant SNPs controlling YPP of the LPS population explained 16.58% and 16.51% of phenotypic variance in the Chang7-2 and PH6WC TC populations, respectively (Figure 5b). The analysis proved that the significant SNPs controlling LPS performance also controlled TC performance.

We further compared the locations of significant SNPs detected for each trait. Two common QTLs on chromosomes 4 and 6 were associated with HKW of the LPS, Chang7-2 and PH6WC TC populations. In addition, one QTL at the end of chromosome 6 was commonly detected in LPS and Chang7-2 TC populations; three common QTLs on chromosomes 2, 3 and 9 were identified in LPS and PH6WC TC populations; and two common QTLs on chromosomes 1 and 7 were identified between Chang7-2 TC and PH6WC TC populations (Figure 2a, Table S2). For YPP, one common QTL on chromosome 1 was detected in LPS and Chang7-2 TC populations, one common QTL on chromosome 2 was detected in LPS and PH6WC TC populations, and two common QTLs on chromosomes 1 and 2 were detected in Chang7-2 and PH6WC TC populations (Figure 2a). The analysis revealed that there were common QTLs between each pair of the three populations, reflecting their strong phenotypic correlations (Figure 5a).

### 2.5. RNA-seq Analysis Identified the Candidate Genes in the Surrounding Region of the Significant SNPs

**Figure 6:**
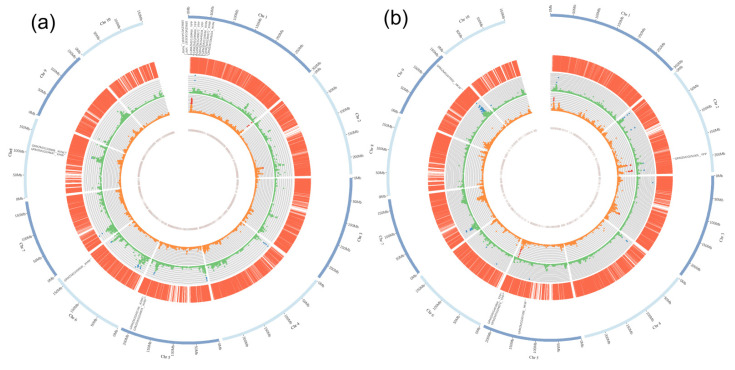
Co-localization of significant SNPs and DEGs identified candidate genes for hybrid performance. (**a**) Co-localization of significant SNPs detected in the Chang7-2 TC population and the DEGs between ZD958 and its parental lines (Zheng58 and Chang7-2). The five circles, from inner to outer, indicate the location of common DEGs identified between ZD958 and each of its parental lines, the MTA results for YPP in the Chang7-2 TC population (red points indicate significant SNPs), the MTA results for HKW in the Chang7-2 TC population (blue points indicate significant SNPs), SNP density heatmap and the candidate genes in the surrounding regions of significant SNPs, and maize chromosomes. (**b**) Co-localization of significant SNPs detected in the PH6WC TC population and the DEGs identified between XY335 and its parental lines (PH6WC and PH4CV). The five circles, from inner to outer, indicate the location of common DEGs between XY958 and each of its parental lines, the MTA results for YPP in the PH6WC TC population (red points indicate significant SNPs), the MTA results for HK in the PH6WC TC population (blue points indicate significant SNPs), SNP density heatmap and the candidate genes in the surrounding regions of significant SNPs, and maize chromosomes.

To find the candidate genes associated with hybrid performance of Chang7-2 TC lines, we found common DEGs between ZD958 and each of its parents (Zheng58 and Chang7-2) and compared the locations of these DEGs with those of significant SNPs. RNA-seq analysis identified 4593 common DEGs (Figure 6a). According to the candidate genes found in our previous article, the orthologs of 57 and 102 DEGs (Table S3) were related to the control of kernel weight and yield, respectively [18]. Among the 57 DEGs, the locations of nine DEGs were close to four HKW QTLs detected in the Chang7-2 TC population (Figure 6a, Table S3), including *GRMZM2G159456*, *GRMZM2G399072*, *GRMZM2G445634*, *GRMZM2G420357*, *GRMZM2G034876*, *GRMZM2G092749*, *GRMZM2G059939*, *GRMZM2G328988* and *GRMZM2G034647*. Meanwhile, five of the 102 DEGs were found in the surrounding regions of one YPP QTL detected in the Chang7-2 TC population, including *GRMZM2G095968*, *GRMZM2G159456*, *GRMZM2G399072*, *GRMZM2G445634* and *GRMZM2G420357* (Figure 6a, Table S3).

To find the candidate genes in the surrounding regions of the significant SNPs detected in the PH6WC TC population, we first found 1801 DEGs that were commonly detected between XY335 and each of its parents. Among the 1801 DEGs, 12 and 20 DEGs (Table S4) were related to the control of yield traits [18]. Among the 12 DEGs, two candidate genes (*GRMZM2G007288* and *GRMZM5G875502*) were found in the surrounding regions of two HKW QTLs detected in the PH6WC TC population. Meanwhile, three DEGs (*GRMZM2G050305*, *GRMZM2G034876* and *GRMZM2G463904*) were found in the surrounding regions of two YPP QTLs detected in the PH6WC TC population (Figure 6b, Table S4). The genes mentioned in this section could be considered candidate genes for the QTLs controlling HKW and YPP.

## 3. Discussion

Maize has rich genetic diversity and rapid linkage disequilibrium, and MTA analysis of various traits has been performed in maize [19,20,21,22]. Many methods have been developed to increase the calculation speed and statistical power of MTA, such as the general linear model (GLM), mixed linear model (MLM), etc., but most of them only work for inbred line populations [23]. At present, there are only a few published MTA methods suitable for performing MTA analysis in hybrid populations, including EMMAX software, which uses different encoding schemes to discriminate additive, dominant and recessive effects [10]; the linear mixed model, which fits additive and dominant effects [24]; and the PEPIS pipeline, which contains all genetic effects [12]. However, various genetic effects (additive, dominant and recessive) are dissected in the former two methods, which complicates the results of MTA analysis. Moreover, the epistasis effect is not considered in the former two methods. The PEPIS pipeline comprehensively dissects the main effect and calculates the LRT values of each SNP in a user-friendly manner. Therefore, PEPIS was used for MTA analysis in this study.

Identification of QTLs and candidate genes controlling agronomic traits is the basis for developing functional markers and molecular design breeding in maize [25,26]. Although many QTLs have been identified using family-based QTL mapping or association mapping [18,27], the QTLs or genes identified using family-based or homozygous lines are different from those detected using hybrid populations [26,28], and most of these QTLs were not proven functional in hybrid lines. In this study, we not only detected common QTLs in the LPS and TC populations but also detected specific QTLs in TC populations. The results showed that the genetic basis of LPS and TC populations is not completely different. The two common QTLs for HKW on chromosomes 4 and 6 with effects in all the three populations required further investigation. Although no common QTLs were detected in the three populations for YPP, there are still common QTLs shared between at least two populations, indicating that YPP might have a complex genetic basis [28]. These common QTLs indicated that manipulating QTLs in the LPS population could increase the yield traits of the hybrid population. Additionally, because the four parental lines of the tested populations were parents of the two most popular hybrid varieties in China, the detected QTLs for yield-related traits could explain why the two hybrid varieties are high-yield and popular in China.

Compared with the results of previous studies, it was found that the significant SNPs Chr3_104753320 (on chromosome 3) and Chr4_9699802 (on chromosome 4) associated with HKW were detected in the LPS and TC populations, respectively, which coincides with the results of a previous study [29]. A significant locus, Chr2_130338518, associated with YPP was detected in the LPS population, and this locus was also detected in an RIL population [29]. Because research on genetic mapping is scarce in maize hybrid populations, most QTL detected in this study were specific. This study has some limitations; although some significant epistatic QTLs (additive × additive, additive × dominant and dominant × dominant) were associated with hybrid performance [29,30,31], we only considered the main effect and did not identify their modes of inheritance [12]. However, this drawback did not influence the identification of common QTLs in LPS and TC populations or the process of finding candidate genes by colocalization.

GP has been proven as a reliable method for predicting both LPS and TC performance [32,33]. Because GP relies on the genetic basis of the population [17,34], the distance between the molecular markers and the QTLs of the target traits could influence the PAs of GP models. In this study, we used GP models to prove that the significant SNPs are reliable because the PAs of GP models fitting significant SNPs were larger than those fitting random SNPs [32]. However, the PAs of GP models fitting significant SNPs were lower than those of GP models fitting genome-wide SNPs, indicating that some QTLs were not detected in each population, which might be related to the high threshold level used in MTA analysis. Furthermore, the realness of the detected QTLs was also supported by the results that both HKW and YPP increased with increased favorable genotypes.

RNA sequencing analysis has been used to dissect the genetic basis of crop traits in combination with genetic methods such as association mapping and linkage-based QTL mapping [15,16]. In this study, in order to find candidate genes underlying hybrid performance, we identified the DEGs between F1 and its parental lines and compared the locations of these DEGs with those of the significant SNPs. The DEGs between F1 and the parental lines might be candidate genes, especially the 14 candidate genes for yield traits (Figure 6b, Tables S3 and S4). Among the 14 candidate genes for kernel weight, *GRMZM2G159456*, *GRMZM2G399072*, *GRMZM2G445634*, *GRMZM2G034876*, *GRMZM2G007288* and *GRMZM5G875502* are orthologous genes of rice *BU1* [35], *SNB* [36], *TIFY* [37], *GL2* [38], *GW2* [39] and *OsiEZ1* [40] genes, respectively, which have been reported to regulate rice seed weight; *GRMZM2G420357*, *GRMZM2G092749*, *GRMZM2G059939* and *GRMZM2G034647* are orthologs of Arabidopsis *IKU1* [41], *FERONIA* [42], *DPA4* [43] and *CYCB1:4* [44] genes, respectively, and these genes are associated with *Arabidopsis* seed weight. Furthermore, *GRMZM2G328988*, *GRMZM2G463904*, *GRMZM2G095968* and *GRMZM2G050305* are orthologous to *UPL3* in oilseed rape [45], to *RLK7* in maize [46], to *IbEXP1* in sweet potato [47] and to *GmMYB14* in soybean [48], respectively. These genes were also reported to be associated with seed weight or overall yield. Therefore, the 14 DEGs for kernel weight might be candidate genes because they were close to the positions of the significant SNPs identified in the TC populations.

## 4. Materials and Methods

### 4.1. Population Construction, Phenotype Evaluation and Phenotypic Data Analysis

Four elite inbred lines were used in this study, including Zheng58, Chang7-2, PH6WC and PH4CV. Zheng58 and Chang7-2 were the female and male parents of ZD958, respectively. PH6WC and PH4CV were the female and male parents of XY335, respectively. ZD958 and XY335 are popular hybrid varieties in China [49,50]. The 481 BC1F3:4 families were introduced in detail in [32]. Briefly, PH4CV was used as the recurrent parent, and Zheng58 was used as the donor parent to develop a BC1F3 population, which was self-pollinated to develop the BC1F3:4 families. The BC1F3:4 families were defined as an LPS population. The 481 BC1F3 plants testcrossed to Chang7-2 and PH6WC in the winter of 2015 in Sanya (Hainan province), producing Chang7-2 and PH6WC TC populations, respectively. These materials were frequently used in maize genetic improvement research (https://maizedata.cn/, accessed on 3 October 2021) [33,51,52]. The materials and populations were provided by the molecular genetic improvement group of the Institute of Crop Sciences, Chinese Academy of Agricultural Sciences.

The LPS and TC populations were sown in Shunyi (Beijing municipality) and Changji (Xinjiang Uygur Autonomous region) in the summer of 2016 and 2017. The two TC populations were also sown in Xinxiang (Henan province) in the summer of 2017. The five environments were identified as 16BJ, 17BJ, 16XJ, 17XJ and 17HN, where BJ, XJ and HN indicate Beijing, Xinjiang and Henan locations, respectively, and 16 and 17 indicate the years 2016 and 2017, respectively. The field experimental design was an incomplete block design, as explained in detail in in our previous publication [33]. The row length and row space were 5 m and 60 cm, respectively, and the planting density was 4444 plants per mu, where mu is a traditional Chinese unit for measuring field size. At the harvest stage, the yield of each plot was measured and adjusted to 14% water content. YPP was calculated by dividing the plot yield into the number of plants in the plot. The hundred kernel weight (HKW) of each plot was measured manually. For HKW, the Chang7-2 and PH6WC TC populations were evaluated in five environments (16BJ, 17BJ, 16XJ, 17XJ and 17HN), whereas the LPS population was evaluated in four environments (16BJ, 17BJ, 16XJ and 17XJ). For YPP, the Chang7-2 and PH6WC TC populations were evaluated in four environments (16BJ, 17BJ, 17XJ and 17HN), whereas the LPS population was evaluated in three environments (16BJ, 17BJ and 17XJ). All experimental research on plants, including collection of plant materials, complied with institutional, national or international guidelines. Field studies were conducted in accordance with local legislation. 

The model for calculating BLUEs is as follows [53,54]:

$$y_{ikmb}=\mu +g_{i}+\tau_{k}+g\tau_{ik}+\delta_{\left(k\right)m}+\beta_{\left(m\right)b}+\varepsilon_{ikmb}$$

where $y_{ikmb}$ is the phenotypic data of the $i_{th}$ genotype in the $b_{th}$ block nested in the $m_{th}$ replication that is nested in the $k_{th}$ environment, μ is the overall mean, $g_{i}$ is the genotype effect, $\tau_{k}\;$is the environmental effect, $g\tau_{ik}$ is the G × E effect, $\delta_{\left(k\right)m}\;$is the replication effect nested in each environment,$\;\beta_{\left(m\right)b}$ is the block effect nested in the replication effect, and $\varepsilon_{ikmb}$ is the residual error. When calculating the BLUEs, the other variables (except the genotype) are treated as random effects and assume to follow normal distributions. All factors are random effects when calculating broad-sense heritability; the formula is as follows [55]:

$$H^{2}=\frac{\sigma_{g}^{2}}{\sigma_{g}^{2}+\frac{\sigma_{g\tau }^{2}}{n}+\frac{\sigma_{\varepsilon }^{2}}{n*r}}$$

where $\sigma_{g}^{2}$ is the genotype variance; $\sigma_{g\tau }^{2}$ is the variance of G × E; $\sigma_{\varepsilon }^{2}$ is the error variance; and *n* and *r* are the number of environments and replicates, respectively. The models for calculating BLUEs and broad-sense heritability are fitted by using the lme4 package [56]. The phenotypic data of the LPS and TC populations are available in Table S5.

### 4.2. Genotype Processing

Leaf samples of the 481 BC1F3 plants and two testers (Chang7-2 and PH6WC) were sampled, and DNA of these samples was extracted following the cetyltrimethyl ammonium bromide method [57]. DNA was sent to Beijing Capital Bio for genotyping using DNA chips containing 55,000 SNPs [58]. The genotypic data were filtered by following the steps: (1) SNPs with a calling rate lower than 97% were removed; (2) SNPs with no physical position information were removed; (3) SNPs with a missing rate greater than 5% were removed; (4) SNPs with minor allele frequencies lower than 0.05 were removed; and (5) the missing genotypes were input using the codeGen function of the R package “synbreed”, the method “beagle” was used and the other settings were default [59,60]. The minor and major alleles were coded as 2 and 0, respectively, and the heterozygous genotypes were coded as 1 [60]. The genotypes of the testcross population were deduced from the testers (Chang7-2 and PH6WC) and 481 BC1F3 parents. Because some loci of the BC1F3 population were heterozygous (Figure S2), we deduced the genotype of the testcross population as described by Cui et al. [61]. Assuming the genotype code of a loci is defined as aa = 0, Aa = 1, AA = 2, if the genotype of the tester is aa, the genotypes of the testcross progeny would be 0, 0.5 and 1; if the genotype of the tester is AA, the genotypes of the testcross progenies would be 1, 1.5 and 2. The genotypic data of the LPS, Chang7-2 TC and PH6WC TC populations are available in Table S6.

### 4.3. Marker–Trait Association Analysis and Calculation of PVE

In this study, we used PEPIS software to perform MTA analysis. PEPIS software is one of the few public user-friendly tools for performing genetic mapping of hybrid populations [12]. The PEPIS software package is based on a linear mixed model [62], and the statistical method of PEPIS is as follows:

First, the genotype of individual j in marker k is encoded into two numerical variables:

$$Z_{\mathrm{jk}}=\left\{\begin{array}{c} +1\;A\\ 0\;H\\ -1\;B \end{array}\right.,\mathrm{and}W_{\mathrm{jk}}=\left\{\begin{array}{c} 0\;A\\ 1\;H\\ 0\;B \end{array}\right.,$$

where $Z_{\mathrm{jk}}$ and $W_{\mathrm{jk}}$ are indicators of additive and dominant effects, respectively. A (the first homozygous genotype), H (heterozygous genotype) and B (the second homozygous genotype) indicate genotypes of each marker.

Then, the following statistical model is used:

$$\begin{array}{cc} y=X\beta +\sum_{k=1}^{m} & Z_{k}a_{k}+\sum_{k=1}^{m}W_{k}d_{k}\\ & +\sum_{k=1}^{m-1}\sum_{k^{\prime }=k+1}^{m}\left(Z_{k}\#Z_{k^{\prime }}\right){\left(\mathrm{aa}\right)}_{{\mathrm{kk}}^{\prime }}+\sum_{k=1}^{m-1}\sum_{k^{\prime }=k+1}^{m}\left(Z_{k}\#W_{k^{\prime }}\right){\left(\mathrm{ad}\right)}_{{\mathrm{kk}}^{\prime }}\\ & +\sum_{k=1}^{m-1}\sum_{k^{\prime }=k+1}^{m}\left(W_{k}\#Z_{k^{\prime }}\right){\left(\mathrm{da}\right)}_{{\mathrm{kk}}^{\prime }}+\varepsilon \end{array}$$

where y is the *n* × 1 vector of the phenotypic data (BLUE); Xβ is a non-genetic effect; and $a_{k}$ and $d_{k}$are the additive and dominance effects, respectively. For markers k and $k^{\prime }$, ${\left(\mathrm{aa}\right)}_{{\mathrm{kk}}^{\prime }},{\left(\mathrm{ad}\right)}_{{\mathrm{kk}}^{\prime }},{\left(\mathrm{da}\right)}_{{\mathrm{kk}}^{\prime }},{\left(\mathrm{dd}\right)}_{{\mathrm{kk}}^{\prime }}$ are additive × additive, additive × dominant, dominant × additive and dominant × dominant epistatic effects, respectively [63]. For each population, we first constructed the additive genotype matrix and the dominance genotype matrix. Then, we input the two matrices and BLUE data into PEPIS software to run marker–trait association analysis (http://bioinfo.noble.org/PolyGenic_QTL, accessed on 7 June 2019). The LRT threshold for declaring significance was −log10(0.05/the number of markers) according to the PEPIS pipeline [12]. The LRT threshold should be 5.49, given that the number of markers is 15,386. The SNPs with LRT values over 5.49 were identified as significant SNPs.

To calculate the phenotypic variance explained by SNPs (PVE), the significant SNPs were fitted in a multiple linear model [64], from which SSreg and SStol for each SNP were computed. SSreg is the sum of square of each SNP, whereas SStol is the sum of square of the linear model. The PVE of each SNP was calculated by dividing SSreg into SStol.

### 4.4. GP and MAS Analysis

We used the ridge regression best linear unbiased prediction (rrBLUP) model to run GP analysis. The rrBLUP model is [65]:

y=Xβ+Zμ+ε

where y is the BLUEs, β is a vector of the fixed effects including only the overall mean, u is the vector of random effects including only additive effect, ε is the residual error, X and Z are the design matrices. GP was implemented by running five-fold cross validation for 200 repeats. The effects of genome-wide markers were estimated, and the predicted phenotypic values were calculated by inputting the effects of genome-wide markers into the rrBLUP model. PA was calculated as the Pearson correlation coefficient between the observed and predicted phenotype. The R package rrBLUP was used to implement the GP model [66], and the code is available in File S1.

To calculate the PA of MAS model fitting the significant SNPs (defined as MAS.Sig model), a multiple regression model was fitted using the lm function in R. The phenotype was predicted using the predict function [53]. The PA was calculated by running five-fold cross validation for 200 repeats. In order to prove the effect of the MAS model fitting significant SNPs, we also calculated the PA of the MAS model fitting the same number of randomly selected SNPs. The model was defined as the MAS.Random model. We used Wilcox.test to compare the differences among the PAs of the three prediction models (GP, MAS.Sig and MAS.Random models).

### 4.5. Identification of Common QTLs among LPS and two TC Populations

Previously, all QTLs within a 20 cM interval were considered a single QTL [67]. According to a previous report, the average recombination rate was 1 cM/Mb [68], which is approximately 1 Mb. Therefore, we defined SNPs within 20 Mb as in linkage with one QTL.

### 4.6. RNA-seq Analysis and Identification of Differentially Expressed Genes around Significant SNPs

The six materials, including two hybrids (ZD958 and XY335), and their parents (Zheng58, Chang7-2, PH6WC and PH4CV) were sown in July 2018 in Haidian, Beijing. Decapitated shoot tips at the V7 stage were used for RNA extraction. For each material, three biological replicates were used, with each replicate containing three samples. We extracted RNA using an RNeasy plant mini kit (Qiagen, Germany) and checked RNA purity using a kaiaoK5500 spectrophotometer (Kaiao, Beijing, China). Then, we assessed RNA integrity and concentration using an RNA Nano 6000 assay kit of a Bioanalyzer 2100 system (Agilent Technologies, Santa Clara, CA, USA). After purifying mRNA from total RNA using poly-T oligo-attached magnetic beads, we generated the sequencing libraries using NEBNext^®^ Ultra™ Directional RNA Library Prep Kit for Illumina (NEB, Ispawich, MA, USA). The libraries were sequenced using the Illumina Novaseq system with a read length of 150 bp (pair end) at Annoroad Gene Technology (Beijing, China).

The RNA sequencing data were analyzed according to the procedure used in our laboratory [69]. Briefly, the raw data were filtered to remove low-quality reads, adaptor-polluted reads and reads with more than 5% N bases. The filtered clean reads were mapped to the B73 RefGen_V3 genome (www.maizegdb.org, accessed on 5 August 2019) using Hisat2 with default settings. The expression level of each sample was estimated using FPKM (fragments per kilobase of transcripts per million fragments mapped), which is calculated by normalizing raw reads. The threshold for identifying the DEGs was false discovery rate (FDR, *p* value < 0.05), which was computed by using Cufflinks. The code is available in File S1.

Some studies have shown that differentially expressed genes between hybrids and their parental lines contribute to hybrid performance [70,71,72,73]. Therefore, we first identified the DEGs between ZD958 and its parental lines (Zheng58 and Chang7-2) and between XY335 and its parental lines (PH6WC and PH4CV). For the Chang7-2 TC population, the physical positions of common DEGs between ZD958 and each of its parents were compared to those of the significant SNPs identified in the Chang7-2 TC population. Those DEGs located within 20 Mb of significant SNPs were identified as potential candidate genes. In the same way as stated above, common DEGs between XY335 and each of its parental lines were identified, and the physical positions of these DEGs were compared to those of the significant SNPs identified in the PH6WC TC population. In the same way, those DEGs located within 20 Mb of significant SNPs were identified as potential candidate genes. To prioritize the candidate genes for HKW, the DEGs in the surrounding regions of significant SNPs associated with HKW were compared to the candidate genes for kernel weight [18]. In the same way, in order to prioritize the candidate genes for YPP, the DEGs in the surrounding regions of significant SNPs associated with YPP were compared to the candidate genes for yield and yield-related traits [18].

## Figures and Tables

**Table 1:** Basic statistical analysis of HKW and YPP of the three populations.

Trait	Population	Mean ± SD (g)	N	CV (%)	Range (g)	*H*^2^ (%)
HKW	LPS	28.88 ± 2.45	481	8.49	21.73–35.52	82.47
	Chang7-2	31.94 ± 1.22	481	3.81	28.43–35.30	79.07
	PH6WC	36.96 ± 1.33	481	3.59	33.09–41.41	78.91
YPP	LPS	101.50 ± 16.52	475	16.27	52.23–181.65	61.63
	Chang7-2	169.56 ± 9.13	469	5.38	145.28–202.65	55.09
	PH6WC	179.58 ± 9.14	475	5.09	154.86–204.93	58.27

SD—standard deviation, N—population size, CV—coefficient of variance, *H*^2^—broad-sense heritability.

## Data Availability

The phenotype and genotype data used in this study are available as supplementary files. The RNA-seq data were deposited in NCBI, (BioProject ID PRJNA766146). The code is available in File S1.

## Supplementary Materials

The following supporting information can be downloaded at: https://www.mdpi.com/article/10.3390/ijms23095074/s1.

## Abbreviations

GP: genomic prediction; MAS: marker-assisted selection; QTL: quantitative trait locus/loci; SNP: single-nucleotide polymorphism; DEG: differentially expressed genes; HKW: hundred kernel weight; YPP: yield per plant; LPS: line per se; TC: testcross; rrBLUP: ridge regression best linear unbiased prediction; PA: predication accuracy; BJ: Beijing; XJ: Xinjiang; HN: Henan; BLUE: best linear unbiased estimation; LRT: likelihood ratio test; PVE: phenotypic variance explained by SNPs; ANOVA: analysis of variance.

## References

[1] Tilman D., Balzer C., Hill J., Befort B.L. (2011). Global food demand and the sustainable intensification of agriculture. Proc. Natl. Acad. Sci. USA.

[2] Duvick D.N. (2001). Biotechnology in the 1930s: The development of hybrid maize. Nat. Rev. Genet..

[3] Bekavac G., Purar B., Jockovic D. (2008). Relationships between line per se and testcross performance for agronomic traits in two broad-based populations of maize. Euphytica.

[4] Mihaljevic R., Schon C.C., Utz H.F., Melchinger A.E. (2005). Correlations and QTL correspondence between line per se and testcross performance for agronomic traits in four populations of European maize. Crop Sci..

[5] Smith O.S. (1986). Covariance between line per se and testcross performance. Crop Sci..

[6] Schwegler D.D., Gowda M., Schulz B., Miedaner T., Liu W.X., Reif J.C. (2014). Genotypic correlations and QTL correspondence between line per se and testcross performance in sugar beet (*Beta vulgaris* L.) for the three agronomic traits beet yield, potassium content, and sodium content. Mol. Breed..

[7] Pace J., Gardner C., Romay C., Ganapathysubramanian B., Lubberstedt T. (2015). Genome-wide association analysis of seedling root development in maize (*Zea mays* L.). BMC Genom..

[8] Sanchez D.L., Liu S.S., Ibrahim R., Blanco M., Lubberstedt T. (2018). Genome-wide association studies of doubled haploid exotic introgression lines for root system architecture traits in maize (*Zea mays* L.). Plant Sci..

[9] Zaidi P.H., Seetharam K., Krishna G., Krishnamurthy L., Gajanan S., Babu R., Zerka M., Vinayan M.T., Vivek B.S. (2016). Genomic regions associated with root traits under drought stress in tropical maize (*Zea mays* L.). PLoS ONE.

[10] Tibbs C.L., Zhang Z., Yu J. (2021). Status and prospects of genome-wide association studies in plants. Plant Genome.

[11] Huang X., Yang S., Gong J. (2015). Genomic analysis of hybrid rice varieties reveals numerous superior alleles that contribute to heterosis. Nat. Commun..

[12] Liu F., Zhao Y., Beier S. (2020). Exome association analysis sheds light onto leaf rust (*Puccinia triticina*) resistance genes currently used in wheat breeding (*Triticum aestivum* L.). Plant Biotechnol. J..

[13] Zhang W.C., Dai X.B., Wang Q.S., Xu S.Z., Zhao P.X. (2016). PEPIS: A pipeline for estimating epistatic effects in quantitative trait locus mapping and genome-wide association studies. PLoS Comput. Biol..

[14] Liu S., Yeh C.T., Tang H.M., Nettleton D., Schnable P.S. (2012). Gene mapping via bulked segregant RNA-Seq (BSR-Seq). PLoS ONE.

[15] Zhang H., Wang X., Pan Q., Li P., Liu Y., Lu X., Zhong W., Li M., Han L., Li J. (2019). QTG-Seq accelerates QTL fine mapping through QTL partitioning and whole-genome sequencing of bulked segregant samples. Mol. Plant.

[16] Yang Y., Ma Y.T., Liu Y.Y., Lyle D., Li D.D., Wang P.X., Xu J.L., Zhen S.H., Lu J.W., Peng Y.L. (2021). Dissecting the genetic basis of maize deep-sowing tolerance by combining association mapping and gene expression analysis. J. Integr. Agric..

[17] Liu X., Wang H., Wang H., Guo Z., Xu X., Liu J., Wang S., Li W.X., Zou C., Prasanna B.M. (2018). Factors affecting genomic selection revealed by empirical evidence in maize. Crop J..

[18] Zhang H., Lu Y., Ma Y., Fu J., Wang G. (2021). Genetic and molecular control of grain yield in maize. Mol. Breed..

[19] Andre B., Zheng P., Luck S., Shen B., Meyer D.J., Li B., Tingey S., Rafalskl A. (2008). Whole genome scan detects an allelic variant of *fad2* associated with increased oleic acid levels in maize. Mol. Genet. Genome.

[20] Kump K.L., Bradbury P.J., Wisser R.J. (2011). Genome-wide association study of quantitative resistance to southern leaf blight in the maize nested association mapping populating. Nat. Genet..

[21] Li L., Hao Z., Li X. (2011). An analysis of the poly morghisms in a gene for being involved in drought tolerance in maize. Genetics.

[22] Tian F., Bradbury P.J., Brown P.J., Hung H., Sun Q., Flint-Garcia S., Rocheford T.R., Mcmullen M.D., Holland J.B., Buckler E.S. (2011). Genome-wide association study of leaf architecture in the maize nested association mapping population. Nat. Genet..

[23] Yu J., Pressoir G., Briggs W.H., Vroh B.I., Yamasaki M., Doebley J.F., McMullen M.D., Gaut B.S., Nielsen D.M., Holland J.B. (2006). A unified mixed-model method for association mapping that accounts for multiple levels of relatedness. Nat. Genet..

[24] Zhao Y.S., Gowda M., Wurschum T., Longin C.F.H., Korzun V., Kollers S., Schachschneider R. (2013). Dissecting the genetic architecture of frost tolerance in Central European winter wheat. J. Exp. Bot..

[25] Wu L., Han L.Q., Li Q., Wang G.Y., Zhang H.W., Li L. (2021). Using interactome big data to crack genetic mysteries and enhance future crop breeding. Mol. Plant..

[26] Xiao Y.J., Jiang S.Q., Cheng Q., Wang X.Q., Yan J., Zhang R.Y., Qiao F., Ma C., Luo J.Y., Li W.Q. (2021). The genetic mechanism of heterosis utilization in maize improvement. Genome Biol..

[27] Liang Y.M., Liu H.J., Yan J.B., Tian F. (2021). Natural variation in crops, realized understanding continuing promise. Ann. Rev. Plant Biol..

[28] Frascaroli E., Canè M.A., Pè M.E. (2009). QTL detection in maize testcross progenies as affected by related and unrelated testers. Theor. Appl. Genet..

[29] Li D., Zhou Z., Lu X. (2021). Genetic dissection of hybrid performance and heterosis for yield-related traits in maize. Front. Plant Sci..

[30] Jiang L., Ge M., Zhao H., Zhang T. (2015). Analysis of heterosis and quantitative trait loci for kernel shape related traits using triple testcross population in maize. PLoS ONE.

[31] Zhang Z., Liu Z., Hu Y. (2014). QTL analysis of kernel-related traits in maize using an immortalized F2 population. PLoS ONE.

[32] Li D., Xu Z., Gu R., Wang P., Lyle D., Xu J., Zhang H., Wang G. (2019). Enhancing genomic selection by fitting large-effect SNPs as fixed effects and a genotypeby-environment effect using a maize BC1F3, 4 population. PLoS ONE.

[33] Li D., Xu Z., Gu R., Wang P., Xu J., Du D., Fu J., Wang J., Zhang H., Wang G. (2021). Genomic prediction across structured hybrid populations and environments in maize. Plants.

[34] Liu X., Hu X., Li K., Liu Z., Wu Y., Wang H., Huang C. (2020). Genetic mapping and genomic selection for maize stalk strength. BMC Plant Biol..

[35] Tanaka A., Nakagawa H., Tomita C., Shimatani Z., Ohtake M., Nomura T., Jiang C.J., Dubouzet J.G., Kikuchi S., Sekimoto H. (2009). *BRASSINOSTEROID UPREGULATED1*, encoding a Helix-Loop-Helix protein, is a novel gene involved in brassinosteroid signaling and controls bending of the lamina joint in rice. Plant Physiol..

[36] Ma X.S., Feng F.J., Zhang Y., Elesawi E.E., Xu K., Li T.F., Mei H.W., Liu H.Y., Gao N.N., Chen C.L. (2019). A novel rice grain size gene *OsSNB* was identified by genome-wide association study in natural population. PLoS Genet..

[37] Hakata M., Kuroda M., Ohsumi A., Hirose T., Nakamura H., Muramatsu M., Ichikawa H., Yamakawa H. (2012). Overexpression of a rice TIFY gene increases grain size through enhanced accumulation of carbohydrates in the stem. Biosci. Biotechnol. Biochem..

[38] Che R.H., Tong H.N., Shi B.H., Liu Y.Q., Fang S.R., Liu D.P., Xiao Y.H., Hu B., Liu L.C., Wang H.R. (2016). Control of grain size and rice yield by *GL2*-mediated brassinosteroid responses. Nat. Plants.

[39] Hao J.Q., Wang D.K., Wu Y.B., Huang K., Duan P.G., Li N., Xu R., Zeng D.L., Dong G.J., Zhang B.L. (2021). The *GW2-WG1-OsbZIP47* pathway controls grain size and weight in rice. Mol. Plant.

[40] Na J.K., Seo M.H., Moon S.J., Yoon I.S., Lee Y.H., Kim J.K., Lee K.O., Kim D.Y. (2013). N-terminal region of rice polycomb group protein OsEZ1 is required for OsEZ1–OsFIE2 protein interaction. Plant Biotechnol. Rep..

[41] Wang A.H., Garcia D., Zhang H.Y., Feng K., Chaudhury A., Berger F., Peacock W.J., Dennis E.S., Luo M. (2010). The VQ motif protein IKU1 regulates endosperm growth and seed size in Arabidopsis. Plant J..

[42] Yu F., Li J., Huang Y., Liu L., Li D.P., Chen L.B., Luan S. (2014). FERONIA receptor kinase controls seed size in Arabidopsis thaliana. Mol. Plant.

[43] Zhang Y., Du L., Xu R., Cui R., Hao J., Sun C., Li Y. (2015). Transcription factors SOD7/NGAL2 and DPA4/NGAL3 act redundantly to regulate seed size by directly repressing *KLU* expression in Arabidopsis thaliana. Plant Cell.

[44] Ren D.Q., Wang X.C., Yang M., Yang L., He G.M., Deng X.W. (2019). A new regulator of seed size control in Arabidopsis identified by a genome-wide association study. N. Phytol..

[45] Miller C., Wells R., Mckenzie N., Trick M., Ball J., Fatihi A., Du-Breucp B., Chardot T., Lepiniec L., Bevan M. (2019). Variation in expression of the HECT E3 ligase UPL3 modulates LEC2 Levels, seed size, and crop yields in Brassica napus. Plant Cell.

[46] He C.M., Wang J., Dong R., Guan H.Y., Liu T.S., Liu C.X., Liu Q., Wang L.M. (2020). Overexpression of an antisense RNA of maize receptor-like kinase gene ZmRLK7 enlarges the organ and seed size of transgenic Arabidopsis plants. Front. Plant Sci..

[47] Noh S.A., Lee H.S., Kim Y.S., Paek K.H., Shin J.S., Bae J.M. (2013). Down-regulation of the IbEXP1 gene enhanced storage root development in sweetpotato. J. Exp. Bot..

[48] Chen L.M., Yang H.L., Fang Y.S., Guo W., Chen H.F., Zhang X.J., Dai W.J., Chen S.L., Hao Q.N., Yuan S.L. (2020). Overexpression of GmMYB14 improves high-density yield and drought tolerance of soybean through regulating plant architecture mediated by the brassinosteroid pathway. Plant Biotechnol. J..

[49] Ma J., Zhang D.F., Cao Y.Y., Wang L.F., Li J.J., Lubberstedt T., Wang T.Y., Li Y., Li H.Y. (2018). Heterosis-related genes under different planting densities in maize. J. Exp. Bot..

[50] Song W., Shi Z., Xing J.F., Duan M.X., Su A.G., Li C.H., Zhang R.Y., Zhao Y.X., Luo M.J., Wang J.D. (2017). Molecular mapping of quantitative trait loci for grain moisture at harvest in maize. Plant Breed..

[51] Wang J.J., Zhang L., Liu X.J., Wang Z.H. (2013). Preliminary assessment of breeding potential of two exotic populations in improving Xianyu. Guizhou Agric. Sci..

[52] Zhang X.G., Ma C.C., Wang X.Q., Wu M.B., Shao J.K., Huang L., Yuan L., Fu Z.Y., Li W.H., Zhang X.H. (2021). Global transcriptional profiling between inbred parents and hybrids provides comprehensive insights into ear-length heterosis of maize (*Zea mays*). BMC Plant Bio..

[53] Hadasch S., Simko I., Hayes R.J., Ogutu J.O., Piepho H.P. (2016). Comparing the predictive abilities of phenotypic and marker-assisted selection methods in a biparental lettuce population. Plant Genome.

[54] Yang J., Mezmouk S., Baumgarten A. (2017). Incomplete dominance of deleterious alleles contributes substantially to trait variation and heterosis in maize. PLoS Genet..

[55] Hallauer A.R., Carena M.J., Miranda Filho J.B. (2010). Quantitative Genetics in Maize Breeding.

[56] Bates D., Machler M., Bolker B.M., Walker S.C. (2015). Fitting linear mixed-effects models using lme. J. Stat. Soft..

[57] Senior M.L., Heun M. (1993). Mapping maize microsatellites and polymerase chain reaction confirmation of the targeted repeats using a CT primer. Genome.

[58] Xu C., Ren Y.H., Jian Y.Q., Guo Z.F., Zhang Y., Xie C.X., Fu J.J., Wang H.W., Wang G.Y., Xu Y.B. (2017). Development of a maize 55 K SNP array with improved genome coverage for molecular breeding. Mol. Breed..

[59] Browning B.L., Browning S.R. (2009). A unified approach to genotype imputation and haplotype-phase inference for large data sets of trios and unrelated individuals. Am. J. Hum. Genet..

[60] Wimmer V., Albrecht T., Auinger H.J., Schon C.C. (2012). Synbreed: A framework for the analysis of genomic prediction data using R. Bioinformatics.

[61] Cui Y., Li R., Li G., Zhang F., Zhu T., Zhang Q., Ali J., Li Z., Xu S. (2019). Hybrid breeding of rice via genomic selection. Plant Biotechnol. J..

[62] Xu S., Zhu D., Zhang Q. (2014). Predicting hybrid performance in rice using genomic best linear unbiased prediction. Proc. Natl. Acad. Sci. USA.

[63] Xu S. (2013). Mapping quantitative trait loci by controlling polygenic background effects. Genetics.

[64] Zhao K., Tung C.W., Eizenga G.C., Wright M.H., Ali M.L., Price A.H., Norton G.J., Islam M.R., Reynolds A., Mezey J. (2011). Genome-wide association mapping reveals a rich genetic architecture of complex traits in Oryza sativa. Nat. Commun..

[65] Meuwissen T.H.E., Hayes B.J., Goddard M.E. (2001). Prediction of total genetic value using genome-wide dense marker maps. Genetics.

[66] Endelman J.B. (2014). Ridge regression and other kernels for genomic selection with R package rrBLUP. Plant Genome.

[67] Elisabetta F., Maria-Angela C., Pierangelo L., Giorgio P., Luca G., Marzio V., Michele M., Mario-Enrico P. (2007). Classical genetic and quantitative trait loci analyses of heterosis in a maize hybrid between two elite inbred lines. Genetics.

[68] Pan Q.C., Li L., Yang X.H., Tong H., Xu S.T., Li Z.G., Li W.Y., Muehlbauer G.J., Li J.S., Yan J.B. (2016). Genome-wide recombination dynamics are associated with phenotypic variation in maize. N. Phytol..

[69] Liu Y., Zhang Z., Fu J., Wang G., Wang J., Liu Y. (2017). Transcriptome analysis of maize immature embryos reveals the roles of cysteine in improving agrobacterium infection efficiency. Front. Plant Sci..

[70] Chen L., Bian J., Shi S. (2018). Genetic analysis for the grain number heterosis of a super-hybrid rice WFYT025 combination using RNA-Seq. Rice.

[71] Howlader J., Robin A.H.K., Natarajan S., Biswas M.K., Sumi K.R., Song C.Y., Park J.I., Nou I.S. (2020). Transcriptome analysis by RNA-Seq reveals genes related to plant height in two sets of parent-hybrid combinations in easter lily (*Lilium longiflorum*). Sci. Rep..

[72] Ren J., Zhang F., Gao F. (2020). Transcriptome and genome sequencing elucidates the molecular basis for the high yield and good quality of the hybrid rice variety Chuanyou. Sci. Rep..

[73] Shahzad K., Zhang X., Guo L. (2020). Comparative transcriptome analysis between inbred and hybrids reveals molecular insights into yield heterosis of upland cotton. BMC Plant Biol..

