# Supplementary material for: Dissection of the Genetic Basis of Yield Traits in Line per se and Testcross Populations and Identification of Candidate Genes for Hybrid Performance in Maize
Source: Int J Mol Sci. 2022 May 3;23(9):5074. doi: 10.3390/ijms23095074 (PMC9102962; doi:10.3390/ijms23095074)
Supplement: Supplementary file 1 [file ijms-23-05074-s001.zip › File S1.pdf]

Genomic prediction

```
rm(list = ls())
library(openxlsx)
library(agricolae)
Genotype=t(read.csv("D:/…/Geno.Impute.csv",row.names=1,check.names=F))
Phenotype=read.csv("D:/…/perseBLUE.csv",row.names=1)
LRT=read.xlsx("D:/…/Main_LRT.xlsx",sheet=1)
perse=perse[rownames(perse.Pheno),]
index=match(PVE.perse$Marker[Index],colnames(perse))
Mark.Sig=perse[rownames(perse.Pheno),index]
head(Mark.Sig)
data=cbind(perse.Pheno,Mark.Sig)
head(data)
n=200
result=matrix(NA,ncol = 3,nrow=n)
for (i in 1:n) {
  print(i)
  y=data$blue
  L=length(y)
  tst=sample(1:L,L*0.2,replace = F)
  train=setdiff(1:L,tst)
  data1=data[train,]
  fit=lm(blue~.,data=data1)
  tstdata=data.frame(Mark.Sig[tst,])
  Predict=predict(fit,tstdata)
  result[i,1]=cor(as.numeric(Predict),y[tst],use="na.or.complete")
  L.1=setdiff(1:15386,index)
  Random=sample(L.1,length(Index),replace = F)
  Marker.Random=perse[,Random]
  data2=cbind(perse.Pheno,Marker.Random)
  data3=data2[train,]
  fit.2=lm(blue~.,data=data3)
  tstdata=data.frame(Marker.Random[tst,])
  Predict=predict(fit.2,tstdata)
  result[i,2]=cor(as.numeric(Predict),y[tst],use="na.or.complete")
  y.train=y[train]
  fit.3=rrBLUP::mixed.solve(y.train,Z=perse[train,])
  ME.2=as.matrix(fit.3$u)
  y.predict.2=perse[tst,]%*%ME.2+as.vector(fit.3$beta)
  result[i,3]=cor(y.predict.2,y[tst],use="na.or.complete")
}
colnames(result)=c("MAS.lm","MAS.Random.lm","GS")
rownames(result)=rep("Rep",n)
```

```
write.csv(result,"D:/…/result.perse.csv")
Name=c(rep("MAS.lm",n),rep("MAS.Random",n),rep("GS",n))
Value=c(result[,1],result[,2],result[,3])
Rep=rep(c("Rep1","Rep2","Rep3"),n)
```

#####RNA-seq pipeline

```
## download gff3 of maize and convert gff3 file into gtf file
gffread Zea_mays.AGPv3.27.gff3 -T -o Zea_mays.AGPv3.27.gtf

## extract_exons.py of hisat2 extracts exons
extract_exons.py Zea_mays.AGPv3.27.gtf  > Zea_mays.AGPv3.27.exons

## extract_splice_sites.py of hisat2 extracts splice sites
extract_splice_sites.py Zea_mays.AGPv3.27.gtf > Zea_mays.AGPv3.27.splicesites

## hisat2-build for index
hisat2-build -p 8 Zea_mays.AGPv3.27.dna.toplevel.fa --exon Zea_mays.AGPv3.27.exons --ss Zea_mays.AGPv3.27.splicesites Zea_mays.AGPv3.27_tran

hisat2 -x Zea_mays.AGPv3.27_tran --rna-strandness RF -1 path/to/cleandata/D2_1_input_CleanData_R1.fastq.gz -2 path/to/cleandata/cleandata/D2_1_input_CleanData_R2.fastq.gz -S path/to/sam/D2_1_input.sam
samtools view -bS 1--6_1_input.sam | samtools view -h -q 50 -> 1--6_1_input.filtered.bam

samtools sort -T tmp.bam 1--6_1_input.filtered.bam -o 1--6_1_input_pos_sorted.bam

htseq-count -f bam -r pos -s n -a 10 -m union --nonunique none 1--6_1_input_pos_sorted.bam Zea_mays.AGPv3.27.gtf > 1--6_1_input.htseq.count.pos.sorted.txt

rm(list=ls())
library(DESeq2)
setwd("D:/../RNA-seq ")

count <- NA
for (i in c("1--6_1","1-54_1","1-48_1","1-98_1","1-16_1","1-43_1","1-46_1","1-67_1","1-187r1_1","1-187r2_1","1-417r1_1","1-417r2_1")) {
  ls <- read.table(paste(i,"_input.htseq.count.pos.sorted.txt",sep = ""),header = F,stringsAsFactors = F,row.names = 1)
  count <- cbind(count,ls)
```

```
}
counts <- count[,-1]
names(counts) <-
c("1--6_1","1-54_1","1-48_1","1-98_1","1-16_1","1-43_1","1-46_1","1-67_1","1-187r1_1","1-187r2_1","1-417r1_1","1-417r2_1")
countdata <- as.matrix(counts[-c(46431:46435),])

coldata <- read.table("D:/…/sample.txt",sep=" ",header=TRUE,row.names=1)

dds <- DESeqDataSetFromMatrix(countData=countdata,
                              colData=coldata,
                              design = ~ condition)
## calculate FPKM
dds <- estimateSizeFactors(dds)
my.counts.normalized <- counts(dds,normalized=TRUE)
write.table(my.counts.normalized,file = "normalized.reads.txt",sep = "\t",quote = F)

## Construct GRangesList from gtf file
library(rtracklayer)
gff <- import("D:/…/Zea_mays.AGPv3.27.gtf","gtf")
gff.flag <- mcols(gff)$type == "exon"
gff.exon <- gff[gff.flag]
genes_list <- split(gff.exon, mcols(gff.exon)$gene_id)

## genes_list (GRangesList object) and dds (DESeqDataSet) has already been ordered by name internally. Both have the same genes and same order.
if (length(rownames(dds)) == sum(rownames(dds) == names(genes_list)))
{print("genes_list and dds have the same gene order")}
rowRanges(dds) <- genes_list
my.fpkm.from_normalized_counts <- fpkm(dds,robust = TRUE)
write.table(my.fpkm.from_normalized_counts,file = "FPKM.from.normalized.reads.txt",sep = "\t",quote = F)

## calculate differential expression
dds <- DESeq(dds)
resultsNames(dds)

resZ58vsZD958 <- results(dds, contrast = c("condition", "Z58","ZD958"), alpha=0.05)
write.csv(resZ58vsZD958,"D:/谱/mayuting/RNA-seq 分析流程/Z58vsZD958.csv",sep=",",quote = F)

resC72vsZD958 <- results(dds, contrast = c("condition", "C72","ZD958"), alpha=0.05)
write.csv(resC72vsZD958,file="C72vsZD958.csv",sep=",",quote = F)
```

```
res335FvsXY335 <- results(dds, contrast = c("condition", "335F","XY335"), alpha=0.05)
write.csv(res335FvsXY335,file="335FvsXY335.csv",sep=",",quote = F)

res335MvsXY335 <- results(dds, contrast = c("condition", "335M","XY335"), alpha=0.05)
write.csv(res335MvsXY335,file="335MvsXY335.csv",sep=",",quote = F)
```
