# Supplementary material for: Dissection of the Genetic Basis of Yield Traits in Line per se and Testcross Populations and Identification of Candidate Genes for Hybrid Performance in Maize
Source: Int J Mol Sci. 2022 May 3;23(9):5074. doi: 10.3390/ijms23095074 (PMC9102962; doi:10.3390/ijms23095074)
Supplement: Supplementary file 1 [file ijms-23-05074-s001.zip › Table S1.pdf]

Table S1 Brief information on the distribution of SNPs across maize genome

Chr	Physical length (Mb)	Number of SNPs	SNP density (SNP/Mb)
1	301.2	2477	8.22
2	236.97	1576	6.65
3	232.11	1677	7.22
4	246.36	1812	7.36
5	217.64	1511	6.94
6	169.23	1358	8.02
7	175.27	1236	7.05
8	175.79	1311	7.46
9	155.98	1372	8.8
10	150.02	1056	7.04
Mean			7.48
