# Supplementary figures and images for: Dissection of the Genetic Basis of Yield Traits in Line per se and Testcross Populations and Identification of Candidate Genes for Hybrid Performance in Maize
Source: Int J Mol Sci. 2022 May 3;23(9):5074. doi: 10.3390/ijms23095074 (PMC9102962; doi:10.3390/ijms23095074)

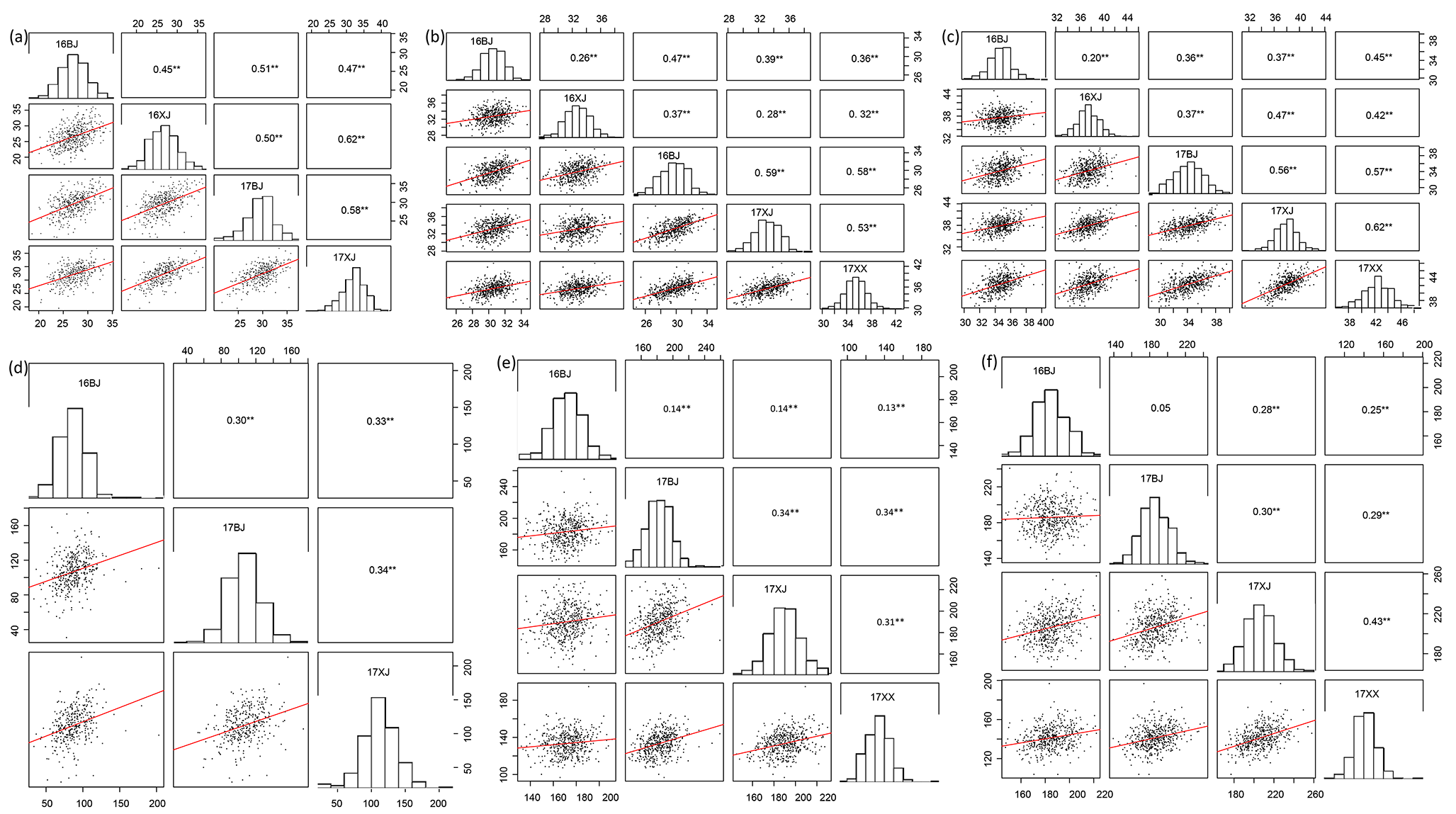

Supplement: Supplementary file 1 [file ijms-23-05074-s001.zip › Figire.S1.tif]

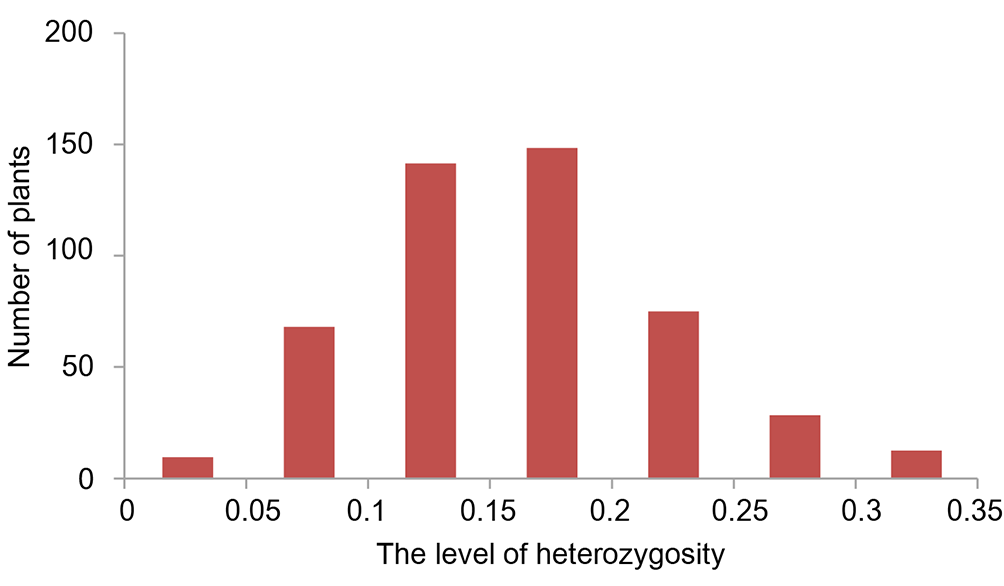

Supplement: Supplementary file 1 [file ijms-23-05074-s001.zip › Figure.S2.tif]
